# Dental Follicle Cells: Roles in Development and Beyond

**DOI:** 10.1155/2019/9159605

**Published:** 2019-09-15

**Authors:** Tao Zhou, Jinhai Pan, Peiyao Wu, Ruijie Huang, Wei Du, Yachuan Zhou, Mian Wan, Yi Fan, Xin Xu, Xuedong Zhou, Liwei Zheng, Xin Zhou

**Affiliations:** ^1^West China School of Stomatology, Sichuan University, Chengdu, Sichuan 610041, China; ^2^State Key Laboratory of Oral Diseases, National Clinical Research Center for Oral Diseases, Department of Pediatric Dentistry, West China Hospital of Stomatology, Sichuan University, Chengdu, Sichuan 610041, China; ^3^State Key Laboratory of Oral Diseases, National Clinical Research Center for Oral Diseases, Department of Operative Dentistry and Endodontics, West China Hospital of Stomatology, Sichuan University, Chengdu, Sichuan 610041, China

## Abstract

Dental follicle cells (DFCs) are a group of mesenchymal progenitor cells surrounding the tooth germ, responsible for cementum, periodontal ligament, and alveolar bone formation in tooth development. Cascades of signaling pathways and transcriptional factors in DFCs are involved in directing tooth eruption and tooth root morphogenesis. Substantial researches have been made to decipher multiple aspects of DFCs, including multilineage differentiation, senescence, and immunomodulatory ability. DFCs were proved to be multipotent progenitors with decent amplification, immunosuppressed and acquisition ability. They are able to differentiate into osteoblasts/cementoblasts, adipocytes, neuron-like cells, and so forth. The excellent properties of DFCs facilitated clinical application, as exemplified by bone tissue engineering, tooth root regeneration, and periodontium regeneration. Except for the oral and maxillofacial regeneration, DFCs were also expected to be applied in other tissues such as spinal cord defects (SCD), cardiomyocyte destruction. This article reviewed roles of DFCs in tooth development, their properties, and clinical application potentials, thus providing a novel guidance for tissue engineering.

## 1. Introduction

The dental follicle (DF), a loose ectomesenchymally derived connective tissue surrounding the tooth germ, participates in tooth eruption and contributes extensively to the periodontium by producing osteoblasts, cementoblasts, and periodontal ligament cells (PDLCs) in tooth development. In 2002, dental follicle cells (DFCs) were firstly isolated from the molar region of mice and induced to differentiate toward an osteoblast phenotype *in vitro* with exogenous bone morphogenetic protein 2 (BMP2) [1]. Since then, DFCs were successively reported to differentiate into osteoblasts, cementoblasts, adipocytes, chondrocytes, and neuron-like cells with appropriate induction conditions [2, 3]. These researches suggested the existence of heterogeneous cells in DF; some of which possess two main characteristics of stem cells: multipotent differentiation and self-renewal. Compared with other dental-derived stem cells like dental pulp stem cells (DPSCs), periodontal ligament stem cells (PDLSCs), stem cells from exfoliated deciduous teeth (SHEDs), stem cells from apical papilla (SCAPs), etc., DFCs exhibited robust proliferative capacity, superior pluripotency, and high immunosuppressed effect which favored tissue engineering (see Table 1). Additionally, the ease and high efficiency of isolation and unrelated ethical issues in clinical contributed to a great feasibility for the application of DFC-based therapy. In this article, we reviewed amounts of recent researches about DFCs focusing on their roles in tooth development, characteristics of multipotent differentiation, and immunosuppressed and excellent proliferation properties, as well as the clinical application advances based on these characteristics; therefore, obtaining a comprehensive recognition of DFCs and providing theoretical and experimental basis to favor DFCs-based treatment in tissue repairment and regeneration.

## 2. DFCs in Tooth Development

Tooth development initiates from the reciprocal interaction between oral epithelium and neural crest-derived mesenchyme, then develops into integral tooth morphogenesis consisting dental crown and root after bud stage, cap stage, and bell stage. The DF starts from the condensed mesenchyme adjacent to the tip of the bud and harbors mesenchymal progenitor cells for various differentiated lineages to compose the tooth root-bone interface and coordinate tooth eruption [4, 5]. It is known that appropriate stimulation from Hertwig's epithelial root sheath (HERS) is of great necessity for tooth root development via inducing the growth, differentiation, and immigration of DFCs. Lack of stimulation from HERS inhibited the capability of DFCs to form cementum and PDL-like tissues [6], and any disturbance of HERS formation produced malformed cementum, abnormal secretion and distribution of collagen fibers crucial to PDL attachment and orientation [7]. Alternatively, HERS indirectly induced the formation of cementum by regulating dental papilla differentiation toward osteoblasts to secrete dentin matrix exposure to DFCs [8]. In turn, DFCs and cementoblasts collaboratively induced apoptosis of HERS cells in tooth development (*in vitro*) in a Fas-Fas ligand (FasL) pathway, followed with upregulated Fas expression on HERS cells and FasL expression on DFCs, respectively [9]. As the development progressed, HERS cells became fragmented at the apex of the developing root to allow cementoblasts or fibroblasts derived from DF to establish connection with outer surface of the tooth root. The activation of transcriptional growth factor-*β* (TGF-*β*) signaling induced HERS fragmentation and promoted HERS to form acellular cementum and PDL via epithelial-mesenchymal transition (EMT) [10].

The establishment of tooth root morphogenesis and coordination of tooth eruption associated with DFCs were dependent on an array of growth and transcription factors consisting Gli1, NOTCH, WNT, nuclear factor 1 C-type (Nfic), and TGF-*β* [7, 8], which are critical to form a healthy dentition from primary tooth eruption to permanent dentition establishment [48]. DFCs on the root surface robustly expressed parathyroid hormone-related peptide (PTHrP) during tooth root formation and after tooth eruption, and PTHrP^+^ DFCs differentiated into PDLCs, alveolar cryptal bone osteoblasts, and cementoblasts in acellular cementum [5, 49]. However, a previous study reported that PTHrP inhibited alveolar bone formation by suppressing WNT/*β*-catenin signaling in DFCs, exhibiting upregulated RANKL/OPG ratio which was in favor of osteoclastogenesis [50, 51]. PTHrP^+^ DFCs subgroups also expressed the PTHrP receptor (PPR) plentifully. PPR-deficient DFCs exhibited obviously truncated roots lacking PDL attachment but enhanced cementoblast differentiation, possibly attributed to the cell fate shift to nonphysiological cementoblast-like cells [5, 49]. Additionally, the cementoblast/osteoblast differentiation of DFCs stimulated by HERS was associated with the WNT/*β*-catenin pathway as the WNT3A expressed on HERS increased alkaline phosphatase (ALP) activity [52]. As for tooth eruption, DFCs recruited and activated osteoclasts and DFCs themselves differentiated into osteoblasts to mediate collaboratively bone remodeling and create space for tooth eruption [53]. PTHrP expressed on DFCs inhibited osteogenesis of DFCs but accelerated tooth eruption [51]. Ameloblast-associated protein (OADM) related to mineralization expressed on DFCs in a time-dependent pattern. The gradually increased OADM expression in the early stage of differentiation accelerated osteogenesis to make a normal eruption, and the missing OADM did not influence bone density but resulted in a postponed tooth eruption [54, 55]. Cleidocranial dysplasia (CCD) patients are characterized by delayed tooth eruption due to runt-related transcription factor 2 (*Runx2*) mutation, DFCs-CCD patients displayed significantly lower osteogenic, osteoclast-inductive and matrix-degrading capacities, mechanistically contacted with disturbed RANK/RANKL/OPG signaling and decreased expression of matrix metalloproteinase 9 (MMP9) and MMP2 [56–58].

## 3. DFC Surface Markers

Stem cells retain the ability of self-renewal and multipotent differentiation, and DFCs have been revealed to hold these potentials. Cell surface markers distinctively express among various stem cells and are mainly classified into three types, embryonic stem cell (ESC) markers, mesenchymal stem cell (MSC) markers, and neural stem cell (NSC) markers. Transcriptional factors SOX2, OCT4, and NANOG expressing on hESCs were crucial to maintain undifferentiated pluripotent stem cells. Around 75% DFCs were identified to express OCT4 and SOX2 both in the nucleus and cytoplasm. In the rat model, it displayed that their expression on DFCs were time-independent during development and were upregulated when cocultured with rat dental papilla cells (DPCs) *in vitro* [12, 59]. NANOG was weakly expressed and downregulated gradually in the subsequent passages of DFCs. Alternatively, DFCs expressed a series of MSC surface markers containing NOTCH-1, STRO-1, CD13, CD44, CD73, CD105, CD56, CD271, and HLA-ABC but not hematopoietic stem cell (HSC) markers like CD34, CD45, and CD133 [60, 61]. STRO-1 and CD44 were widely distributed in DFCs, and their expression were downregulated as the passages increased. Therefore, they were the most common surface markers to identify the existence of MSCs in DF [12, 62]. The transmembrane protein NOTCH-1 was important in various cell fate decisions during development and strongly expressed on DFCs. It was reported to promote the capability of self-renewal and proliferation of DFCs by modulating G1/S phase transition and telomerase activity [63]. A recent study also suggested that 90% of cultured DFCs were positive for HLA-ABC which has been reported in PDL and dental pulp [12]. As described previously, DFCs expressed nestin (a neural progenitor cell marker) and beta-III-tubulin (an early neuronal marker), and the presence of neural crest stem cell markers (P75 and HnK1) and glial-like cell markers (GFAP) were also reported in the DF [12, 64, 65].

## 4. Multipotent Differentiation of DFCs

### 4.1. Osteogenic Differentiation

**Figure 1:**
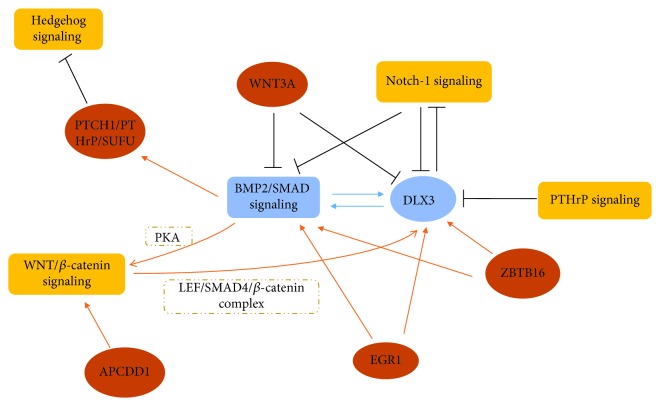
BMP2/DLX3 integrated network in DFC osteogenic differentiation.

DFCs are responsible to form alveolar bone in tooth development to support and fix the tooth root, which are also capable to differentiate into osteoblasts and form mineralized matrix nodules with appropriate exogenous osteogenic stimulus, such as dexamethasone or BMPs [66, 67]. During the osteogenic differentiation, the proteomic analysis suggested that 115 proteins were differentially regulated, in which 80 proteins like glutamine synthetase and beta-actin were upregulated while 35 proteins like cofilin-1 and pro-alpha1 collagen were downregulated [53]. It is elucidated that the expression of osteogenic-related markers including RUNX2, ALP, bone sialoprotein (BSP), and osteocalcin (OCN) were enhanced in this process [68, 69]. In spite of the complex transcriptomic and proteomic changes during osteogenic differentiation, only a minor number of identified proteins could be assigned to specific pathways currently. These transcriptional factors and signaling pathways collaboratively participating osteogenic differentiation of DFCs are mainly in a BMP2/the distal-less homeobox-3 (DLX3) integrated molecular network. Signaling pathways critical to bone formation such as BMP, NOTCH, Hedgehog, WNT signaling, and transcription factors mainly acted on the BMP2/DLX3 feedback loop to perform a positive or negative regulation for DFC osteogenic differentiation (see Figure 1).

#### 4.1.1. BMP Signaling

BMPs are a group of glycoproteins belonging to the TGF-*β* superfamily, and they impact DFC osteogenic differentiation both through the canonical and noncanonical pathways. The signaling transduction of the canonical pathway is initiated when BMPs bind to their receptors BMPRs (BMPR1A, BMPR1B, and ActR-1A) to form a heterotetrameric complex composed of two dimers of type I and type II serine/threonine kinase receptors. Subsequently, phosphorylated BMPs activate SMADs, while the noncanonical pathway is SMAD-independent [70]. More than 20 BMPs are found to modulate osteogenic differentiation directly or act as the intermediate regulator to influence bone formation. BMP2, BMP4, BMP6, BMP7, and BMP9 are five most studied subtypes upon DFC osteogenic differentiation. Though all of them behaved promoted effect on osteogenesis, the mechanisms and effect levels were distinctive. Both BMP2 and BMP7 mediated DFC osteoblast differentiation in a time and dose-dependent manner while others were not [1, 71]. BMP2 and BMP4 were critical to the early stage of osteogenic differentiation while BMP6 functioned both in the early and late stages of this process. Previous evidence supported that high BMP6 expression effectively maintained osteogenic capability of DFCs and exogenous human recombinant (hrBMP6) can partially restore the differentiated capability of DFCs in late passages [72]. Mechanistically, BMP6 enhanced the phosphorylation of SMAD1/5/8 proteins associated with canonical BMP signaling while BMP2 and BMP9 got involved in the MAPK signaling pathway [73, 74]. Secreted BMP6 from other cells in DF also promoted the osteogenesis via a paracrine pathway. Interestingly, BMP6 was also considered as one of the downstream targets of circular RNAs (circRNAs) in DFC differentiation, it was upregulated in circFgfr2-enhanced DFC osteogenesis [75].

In 2012, Viale-Bouroncle et al. firstly put forward the significant status of the BMP2/DLX3 feedback loop in regulating osteogenic differentiation of DFCs. The study displayed that BMP2 induced the expression of DLX, and in turn, DLX3 upregulated BMP2 and activated the BMP/SMAD1 signaling pathway [76]. Furthermore, subsequent evidence supported that BMP/DLX3 acting as the central axis integrated a series of signaling pathways which participated osteogenic differentiation of DFCs. NOTCH-1 expression was regulated as DFCs differentiated and it played a negative role on osteogenic differentiation of DFCs via destroying the expression of DLX3 and activation of SMAD1 [77]. Conversely, DLX3 overexpression enhanced NOTCH signaling in DFCs, displaying a negative feedback regulation. *β*-catenin was phosphorylated via intermediate protein kinase A (PKA) induced by BMP2 and DLX3, then formed the lymphoid enhancer factor (LEF)/SMAD4/*β*-catenin complex to promote DLX3 transcription to facilitate osteogenic differentiation, therefore establishing a cross talk between the WNT/*β*-catenin and BMP2/SMAD signaling pathways. As the canonical WNT signaling induced by WNT3A negatively adjusted DFCs osteogenic differentiation, the BMP2/DLX3-induced PKA/*β*-catenin pathway antagonized the inhibitory effect and sustained differentiation capability to some extent [78]. The hedgehog signaling was greatly required in tooth development but slightly inhibited ALP activity and mineralization of differentiated DFCs. With the induction of BMP2 *in vitro*, the hedgehog signaling was repressed during DFC osteogenic differentiation as its inhibitors patched1 (PTCH1), suppressor of fused (SUFU), and PTHrP were upregulated [79]. Except for the suppressor, PTHrP was also the targeted gene of hedgehog signaling, and it was highly concentrated extracellularly and slightly upregulated intracellularly during the osteogenesis in DFCs. PTHrP overexpression inhibited ALP activity and DLX3 transcription but in a hedgehog-independent way [51, 80]. As PKA was regulated downstream in PTHrP signaling and involved the regulation of DLX3 in DFC differentiation, it may be a targeted intermediate in PTHrP-mediated osteogenesis.

#### 4.1.2. WNT Signaling

WNT signaling is essential to embryonic development which regulates the proliferation, differentiation, migration of stem cells, and the epithelial-mesenchymal interactions critical to dental tissues. WNTs are secreted glycoproteins acting as the ligands to activate the canonical and noncanonical WNT signaling pathways. The activation of canonical signaling is initiated from the binding of the WNT ligand to Frizzled (Frz) protein and coreceptor, then followed with the phosphorylation of the multiprotein complex and increased cytoplasmic and nuclear *β*-catenin level, eventually cooperated with T cell factor (TCF)/LEF transcription factors and other coactivators to regulate the target genes [81]. The crucial protein *β*-catenin expressed in DF and its expression coincided with the period of osteogenesis and cementogenesis. Increased activity of nucleus *β*-catenin and *β*-catenin/TCF luciferase induced by lithium chloride (LiCl) led upregulation of OCN, RUNX2, type I collagen (COLI) proteins, and ALP activity, which suggested a positive role of WNT signaling in the DFC osteogenesis [82]. Adenomatosis polyposis coli downregulated 1 (APCDD1) was crucial for sustaining the expression of *β*-catenin and the activity of the TCF/LEF promoter in DFCs. The deletion of APCDD1 reduced the expression of osteogenic markers and matrix mineralization, and it was also regarded to be involved in BMP2/DLX3-directed osteogenic differentiation through *β*-catenin [83]. The essential ligand WNT3A in WNT signaling impeded mineralized nodule formation and reduced RUNX2, OCN levels, and ALP activity in DFCs, it also suppressed BMP2-induced osteoblast differentiation *in vitro* in a *β*-catenin/TCF-dependent mechanism [84]. We account that WNT3A may exert a double effect as the DFCs mineralization was enhanced when cocultured with HERS expressing WNT3A [8]. The available evidence demonstrated that this dual role of WNT3A was mediated through the WNT signaling pathway. However, it is still unclear whether it is led by different cell types or differentiation stages or even the involvement of other signaling pathways. DKK3, an inhibitor of WNT/*β*-catenin, also negatively regulated osteogenesis of DFCs by influencing formation of calcified nodules [85]. Other proteins also regulated DFCs differentiation indirectly through the WNT/*β*-catenin pathway. The anterograde intraflagellar transport motor KIF3A in primary cilia activated indirectly the WNT/*β*-catenin pathway triggered by WNT3A. Deletion of *Kif3a* resulted the attenuation of active *β*-catenin and LEF1, eventually displayed substantial impairment of mineralization and differentiation-associated marker expression [86]. Naked cuticle homolog 2 (NKD2) has been reported to promote DFCs to differentiate to osteoblasts through WNT/*β*-catenin as a signal-inducible feedback antagonist [87]. From the perspective of epigenetics, the decrease of maternally expressed 3 (MEG3) or enhancer of zeste homolog 2 (EZH2) activated the WNT/*β*-catenin signaling pathway via epigenetically regulating the H3K27me3 level on the WNT gene promotors, which offered a new guideline for osteogenesis research in DFCs [88].

The noncanonical WNT signaling is *β*-catenin independent and is also initiated when WNT ligand binds to Frz and its coreceptor. Then, dishevelled (DSH) is recruited to interact with a series of proteins to activate downstream target molecules like C-Jun N-terminal kinase (JNK). WNT5A mediated the noncanonical WNT signaling pathway and regulated cell proliferation, differentiation, and polarization. WNT5A was expressed in DFCs, especially displayed a robust expression in alveolar bone on postnatal days 1-11. The overexpression of WNT5A in DFCs promoted phosphorylation of JNK1/2, which was similar to that in DPCs and bone marrow stem cells (BMSCs) [89, 90]. Except for acceleration of osteogenesis, WNT5A also took a part in osteoclast lineage by regulating RANKL ligand expression in a positive manner, thus mediating bone resorption and remodeling [91]. Recent evidence demonstrated a complex interaction between canonical and noncanonical WNT signaling in DFC differentiation. Silence of *Wnt5a* in DFCs enhanced WNT3A-mediated increase of ALP expression, while the negative role of WNT5A was not related to nuclear translocation of *β*-catenin and transcriptional activation of TCF triggered by WNT3A. It was considered to inhibit the downstream part of the *β*-catenin/TCF pathway [92].

#### 4.1.3. Transcriptional Factors

In DFC osteogenesis, around 1/3 regulated genes had promoter binding sites for transcriptional factors TP53 and SP1. TP53 overexpression promoted osteogenic differentiation of DFCs while SP1 showed a more obvious impact on DFC proliferation, whereas the involved mechanism was unclear [93]. Besides, zinc factor and BTB domain containing 16 (ZBTB16) performed multiple and complex functions in DFC osteogenic differentiation. It upregulated late osteogenic marker expression like OCN while ALP and RUNX2 were not affected [68]. And dexamethasone-induced DFC osteogenic differentiation was reported in a ZBTB16-dependent manner [94, 95]. It was worth mentioning that ZBTB16 also regulated the BMP2/DLX3 feedback loop as it induced the expression of BMP2 and had a binding site on the DLX3 promoter. Another potential mechanism for ZBTB16 in DFC differentiation may associate with the expression of a new target gene stanniocalcin 1 (STC1) which was responsible for mediating osteogenic-related marker OPN and OCN expression [68]. Early growth response protein1 (EGR1) was critical to proliferation, apoptosis, and differentiation. The level of EGR1 was elevated after osteogenic differentiation of DFCs and in turn it regulated the expression of DLX3 and BMP2 to mediate osteogenic differentiation positively [96]. Fractional odontogenic matrix protein such as dentin matrix protein 1 (DMP1) and odontogenic ameloblast-associated protein (ODAM) have been identified to correlate positively with the osteogenic capability of DFCs, which suggested the complex microenvironment in different time and space for DFC differentiation in tooth development [54, 97].

Except for the signaling pathways and biological factors mentioned above, physical factors covering the temperature, stress, and stiffness also influenced DFC osteogenic differentiation. It was reported that soft extracellular matrix, elevated temperature contributed to the proliferation, differentiation, and expression of related markers of DFCs [98, 99]. The role of cell-cell interaction in the complex development microenvironment recently gained increasing attention, except for HERS, *in vitro* studies suggested that the osteogenesis and fibrogenesis abilities of DFCs were inhibited when cocultured with SCAPs [100]. Alternatively, increased angiogenic activity in DFCs and human umbilical vein endothelial cells (HUVECs) co-cultures stimulated osteoblast maturation of DFCs [101].

### 4.2. Neural Differentiation

The neural lineage differentiation of DFCs is partially attributable to its origin from neural crest. Early studies reported the neural characteristics of DFCs in specific culture conditions, such as the expression of neural markers and the capability to differentiate to functionally active neurons. When placed DFCs into a neutron induction medium, the differentiated multipolar neuron-like cells expressed late neural markers and exhibited capability to produce a sodium current consistent with functional neuronal cells [2, 102]. The glial cell marker glial fibrillary acidic protein (GFAP) was restrictedly expressed on DFCs, which may suggest a limited differentiation potential of DFCs to glial cells. But the glial cell differentiation can be enhanced remarkably via activating the TGF-*β* signaling pathway through the phosphorylation of SMAD2 in DFCs [103]. Compared with DPSCs and SCAPs, DFCs had a higher proliferation capability and expressed upregulated CNPase (a myelin protein expressed both on oligodendrocytes and Schwann cells) and DCX (a specific protein expressed on neuronal cells) in consistent conditions, which supported that DFCs may act as a better candidate type for neural differentiation [104, 105]. In spite of the neural differentiation potential, superior strategies for DFCs to produce both neural-like and functional neuronal cells are challenges in the complex microenvironment in the body. Previous researches imposed a two-step strategy for neuronal differentiation of DFCs *in vitro* including predifferentiation and selective induction. DFCs predifferentiation was performed to obtain neurosphere-like cell clusters (NLCCs) in which neural cell markers like beta-III-tubulin, NSE, and nestin were upregulated. Then, these NLCC-derived cells were cultivated in medium whose surface was modified with laminin and poly-L-ornithine, thus exposing neural-like cell morphology with small neurite-like cell extrusions [65]. In view of the two-dimensional culture medium was hard to mimic the highly complex extracellular matrix (ECM) environment of stem cells undergoing neurogenesis *in vivo*. Researchers considered to use decellularized matrix (DECM) extracted from neural stem cells (NSCs) differentiated from hESCs to simulate the natural physiological microenvironment in DFC neurogenesis. The outcome supported that NSC-DSEM was superior in enhancing DFC neural differentiation [106].

### 4.3. Periodontium Differentiation

One of the most important functions of DFCs is to form good root-bone interface, including PDL, cementum, and alveolar bone. Cementum is mineralized tissue covered in the surface of the tooth root and regulates the physical and chemical interaction between PDL and tooth root. In tooth development, cementogenesis initiates at a root-forming stage when epithelial stimulation from HERS induced differentiation of DFCs into cementoblasts/osteoblasts. A combination of DFCs and HERS implanted in immunocompromised mice enhanced the activity of mineralized tissue-forming cementoblasts obviously [107], part of the mechanism resulted from the production of BMP2, BMP4, and BMP7 synthesized by HERS [108]. The structure of the cementum was resembled with the early woven bone; the induction of DFC osteogenic differentiation was usually followed with the formation of a cementoblast phenotype. For example, RUNX2 critical to osteogenesis was present in early proliferative cementoblasts and its overexpression upregulated the expression of cementoblast-related genes of DFCs correspondingly [3]. Specifically, cementoblasts expressed unique markers like cementum-derived attachment proteins (CAP) which promoted the attachment, proliferation, and differentiation of DFCs [109]. DFCs formed cementum-like matrix and expressed osteopontin (OP) and COLI mRNA when they were transplanted into immunodeficient mice [110, 111]. Odontogenic matrix protein like dentin noncollagenous proteins (dNCPs) and enamel matrix derivatives (EMD) could stimulate DFCs to differentiate cementum-like tissues *in vivo*, and biological activities of EMD were mediated by BMPs [112]. It was also suggested that other factors presented in EMD induced the cementogenesis in a SMAD-independent pathway, such as MAPK signaling [113]. Interestingly, DFCs transplants isolated from human molars at a root-developing stage were able to produce a cementum/PDL-like structure, characterized by a thin layer of cementum-like mineralized tissues and PDL-like collagen fibers connecting with the newly formed cementum [114], which demonstrated a higher activity of DFC differentiation potential in developing stages.

PDL is composed of a fibrous extracellular matrix, including collagens, microfibrils, and proteoglycans to provide resistance against occlusal force and nutrition for the alveolar bone and tooth. DFCs on the surface of hydroxyapatite beads formed fibrous tissues when implanted into immunodeficient mice; meanwhile, they expressed mRNA for BSP, OC, OP, and COLI [111]. Mechanistically, F-spondin expressed on DFCs have been reported to downregulate PDL marker genes through inhibiting TGF-*β* activity, thus suppressing the PDL differentiation of DFCs *in vivo* [115]. In spite of the potential to differentiate PDL-like tissues, it is hard to recover the shape and function of natural PDL utilizing DFCs.

### 4.4. Differentiation into Other Lineages

DFCs were capable of forming adipocytes, and stained adipocytes were observed after placing DFCs in an adipogenic medium for 3 weeks [116]. The transient receptor potential melastatin 4 (TRPM4), an ion channel that controls Ca^2+^ signal was necessary for DFCs adipogenesis while it acted as an inhibitory regulator in osteogenic differentiation [117]. The relatively lower chondrocyte differentiation of DFCs than adipocyte differentiation was also reported in previous studies. Interestingly, with the induction of treated dentin matrix (TDM), DFCs differentiated to odontoblasts to form dentin-like tissues via expressing a higher level of odontogenic markers such DMP-1 and DSP than DPCs [118, 119]. Additionally, a recent study also reported that DFCs differentiated into cardiomyocytes with suberoylanilide hydroxamic acid (SAHA) *in vitro*, which extended the recognition of DFCs [120]. In conclusion, DFCs possessed superior multilineage differentiation capabilities, which provided a significant prerequisite and research foundation for DFC treatment in repairment and regeneration of tissue defects.

## 5. Immunomodulatory Properties of DFCs

In spite of the multidifferentiation of DFCs expected to be used in tissue repairment, damaged or exposed tissue wounds are often accompanied by inflammatory infections which suppressed the differentiation of stem cells. In patients with periodontitis, the complex oral microenvironment accumulating amounts of anaerobic periodontal pathogens and bacterial toxins is the main issue leading to the failure of multiple treatment. Besides, we have to pay attention to the immune responses caused by the proliferation and differentiation of allogeneic cells in cell-based therapy.

**Figure 2:**
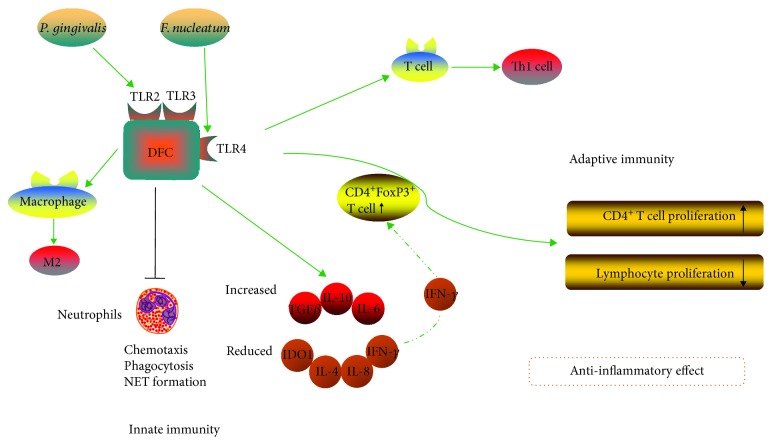
The immunosuppression of DFCs linked with innate and adaptive immunity.

DFCs surface expressed Toll-like receptors (TLR) like TLR2, TLR3, and TLR4. They are a kind of pattern recognition receptors which are broadly distributed on immune system cells to connect innate and adaptive immune responses (see Figure 2). TLR4 can be activated by the lipopolysaccharide (LPS) of gram-negative species such as *F. nucleatum*, while *P. gingivalis* LPS conveyed signals via TLR2 [121, 122]. LPS-pretreated DFCs suppressed peripheral blood mononuclear cell (PBMC) proliferation at the cell ratios, which may be a consequence of significantly downregulated TLR4 in DFCs [121]. In the existence of pathogenic bacterium, DFCs released amounts of cytokines to perform immunomodulation through the innate immune system. When cocultured with lymphocytes from healthy peripheral venous blood, DFCs exhibited decreased IL-4 and IFN-*γ* levels and increased anti-inflammatory cytokine IL-10 [11]. In a cocultured inflammatory environment combining *P. gingivalis* and *F. nucleatum* with DFCs, DFCs behaved higher secretion of IL-10 than proinflammatory cytokine IL-8 at all measured time points and obviously lowered bacterial adherence and internalization capacity [123]. Additionally, after the pretreatment with LPS, it was followed by a higher production of TGF-*β*, anti-inflammatory cytokine IL-6, and reduced indoleamine 2,3-dioxygenase 1 (IDO-1) expression [13]. In comparative studies, LPS from different kinds of pathogenic bacterium behaved distinguished impact on dental stem cells. LPS especially *P. gingivalis* LPS induced the expression of proinflammatory cytokines therefore inhibiting the differentiation of DPSCs [124]. Conversely, the proinflammatory cytokine induction was absent after the administration of *P. gingivalis* LPS in DFCs while *Escherichia coli* LPS induced the expression of IL-6, IL-8, and IL-1*β* in DFCs, thus inhibiting DFC osteoblast differentiation and mineralization [125]. And the inhibition of DFC osteogenic differentiation in an inflammatory microenvironment was related to the increase in TGF-*β*2 levels [126]. This specific reaction may provide a target choice of appropriate cell types for repairment after bacterial culture experiment. Furthermore, DFCs with infection of periodontopathogens behaved a direct impact on chemotactic attraction, phagocytic activity, and NET formation of neutrophils (PMN), reducing PMN-induced tissue and bone degradation via suppression of PMN activity [127]. DFCs also reprogrammed macrophages into the anti-inflammatory M2 phenotype by secreting paracrine factors TGF-*β*3 and TSP-1, which ameliorated LPS-induced inflammation [128].

In addition, DFCs were capable to regulate the adaptive immunity. Cytokines secreted by DFCs exhibited suppressive effect on lymphocyte proliferation and T lymphocyte apoptosis, and the presence of IFN-*γ* strengthened the suppression of DFCs on these cells. Mechanistically, the immunosuppressive effects on lymphocyte proliferation are related to an upregulated frequency FoxP3 which expressed on CD4^+^ CD25^+^ regulatory T cells [11, 129]. Asthma is an allergic disease in which inflammatory responses involve the polarization of CD4^+^ T cells to Th2 cells. The study showed that DFCs exhibited an antiproliferative response to CD4^+^ T lymphocytes by increasing the levels of CD4^+^CD25^+^FoxP3^+^ T regulatory cell frequency and the IDO and TGF-*β* pathways were involved in the induction of T regulatory cells. Besides, DFCs suppressed allergen-induced Th2 cell polarization while supported the differentiation of T lymphocytes toward Th1 cells. In conclusion, the downregulated effect of DFCs on allergen-induced effector, effector memory, and central memory T cell subsets in asthma patients behaved a protective mechanism on naïve T lymphocyte population [130]. Apart from allergic diseases, DFCs were effective to treat autoimmune diseases like MuSK-related myasthenia gravis (MG) through reducing proliferation of lymph node cells and producing IL-6 and IL-12 [131].

## 6. Senescence and Apoptosis Characteristics of DFCs

DF tissue is a potential stem cell bank which can be harvested abundantly from extracted teeth, especially in the case of impacted wisdom teeth extraction. After appropriate isolation procedure and expansion *in vitro*, a sufficient number of DFCs are expected to obtain [132–134]. Unfortunately, even under standard cell culture conditions, DFCs face the challenge of limited cell divisions and enter cellular senescence after a prolonged cell culture [135]. Senescent cells usually behave shortened telomere, changes in morphology and expression of *β*-galactosidase, and the loss of cell proliferation potency [136]. A previous study suggested that DFCs exhibited features of cellular senescence after being expanded after more than 14 cell passages, displaying decreased cell proliferation, enlarged cell size, and upregulated expression of *β*-galactosidase [137]. Short telomeres and increased DNA damage with genomic instability were correlated with the accelerated induction of cellular senescence [138]. Moreover, the osteogenic differentiation of DFCs was inhibited due to cellular senescence, followed with a lower extent to differentiate into biomineralizing cells [137]. During the process of cellular senescence, expression of cyclin-dependent kinase 2 (CDK2) and CDK4 were modulated, and the cell cycle regulatory protein P21, P27, and P18 were all downregulated while P16 was upregulated. The cell cycle protein P16-dependent pathway was considered to drive the induction of cellular senescence of DFCs as the number of senescent cells reduced when P16 gene was silenced [139]. NOTCH signaling was essential to control the proliferation and apoptosis of DFCs. NOTCH-1 signaling regulated the proliferation and self-renewal capacity of DFCs through modulation of the G1/S phase transition and telomerase activity, active NOTCH-1 promoted G1/S transition via decreasing the number of the G1 phase cells and accelerating the S phase transition in DFCs [63, 140]. In addition, NOTCH signaling was elucidated to exhibit a suppressive effect on DFC apoptosis through reducing cytoplasmic apoptotic effects in the classical mitochondrial pathway and the noncanonical NOTCH-1-AKT module, together with repression of *p53* transcription in nuclei [141]. It is worth noting that some biomaterials or chemical substances accelerated the senescence of DFCs. The *β*-tricalcium phosphate (TCP) induced programmed cell death while enhanced bone differentiation, and the survived DFCs exhibited a highly upregulated expression of antiapoptotic genes [142]. Hydroxyurea induced premature via influencing genes associated with DNA damage and repair, mitochondrial dysfunction, and it also increased reactive oxygen species levels. The age was another crucial factor as DFCs from young donors were more resistant to apoptosis and behaved increased nonhomologous end joining activity compared to old donors [143].

## 7. Clinical Application Potential of DFCs

From what were mentioned above, DFCs had multilineage differentiation potential, excellent anti-inflammatory capability, and accessibility to obtain and expand *in vitro*, which lay the foundation for DFC clinical application. To date, the key steps in tissue engineering contain methods of cell isolation, expansion, transplantation, and specific lineage differentiation. The use of bioactive matrix materials such as tissue scaffolds, addition of various hormones, and growth factors or other chemical compounds optimized the strategies in tissue repairment and regeneration. Previous studies have reported the formation of bone-like, PDL-like tissues successfully both *in vitro* and *in vivo* utilizing DFCs combined with various approaches.

### 7.1. Bone Tissue Engineering

Osteogenic differentiation potential makes DFCs an attractive type of stem cells for repairing bone defects or loss caused by periodontal diseases, trauma, or degenerative diseases. Honda et al. initially obtained new bone formation after DFCs transplantation in surgically created calvarial defects in immunosuppressed mice [53]. However, it was difficult to obtain effective tissue repairment relying on cell differentiation singly. Recent views supported a combination of dental stem cells with bioactive materials, an alternative to autologous bone transplants without impairing the proliferation and differentiation of dental stem cells [144]. Early studies considered hydroxylapatite (HAP) and *β*-tricalcium phosphates (TCP) as scaffolds for DFC osteogenic differentiation. TCP was an excellent scaffold for DFC osteogenesis while HAP contributed to a modest differentiation [145]. However, TCP-induced apoptosis of DFCs is unbeneficial for cell-based therapy. A better bone regeneration for healing calvarial critical-size defects was achieved through transplanting DFCs loaded into polycaprolactone (PCL) scaffold that was covered with hyaluronic acid and *β*-TCP. This method promoted cell proliferation, migration, and even dispersion [146]. A novel scaffold composed of biodegradable coralline hydroxyapatite (CHA) seeded with BMP9-transfected rat DFCs (rDFCs) induced new alveolar bone formation. It achieved optimal effects in repairing the alveolar bone defects for forming more new bone and blood vessels, and the osteogenesis was associated with the activation of SMAD1/5/8 signaling induced by BMP9 [147]. Recently, alloplastic materials like titanium and ceramics gained increasingly focus in recovering bone defects. Even without exogenous osteogenic factors like BMP2, titanium with different bioactive coatings was capable of sustaining osteogenic differentiation of DFCs and titanium implants with hydroxyapatite (TiHA) seemed more favorable [148]. To imitate the complex microenvironment *in vivo*, DFCs were precultured from the 5th to 8th passages in a three-dimensional (3D) culture using gelatin sponges and then were transplanted to immunodeficient rats. After 28 days, numerous woven osteoids, enlarged capillary vessels, and spindle-shaped cells were observed and osteoblasts were accumulated around osteoids. Micro-CT as the gold standard for assessing bone morphology and microarchitecture demonstrated a higher quality than the control group [149]. With the advancement of materials science, nanocomposite was applied into tissue engineering, a trilayered nanocomposite hydrogel scaffold implanted into rabbit maxillary periodontal defects with growth factors supported new formation of the alveolar bone [150]. It is also highlighted that nanosilicates with fluoride additive (NS+F) aid evidently enhanced the osteogenic differentiation capabilities of DFCs. Therefore, nanobiomaterials are expected to be a type of a good carrier used for periodontal bone tissue regeneration [151].

### 7.2. Tooth Root Regeneration

Tooth is another type of mineralized tissue in the human body. As DFCs are responsible for forming a tooth root and its supporting tissues in odontogenesis, DFCs were mainly studied to apply in tooth root regeneration. Previous studies isolated DFCs from developing root and loaded them on an absorptive root-shaped scaffold in regular sequence. By this way, they mimicked a biophysiological root *in vivo* and regenerated a functional root/periodontal tissue complex able to support a porcelain crown [114]. The strategy was optimized by combining DFCs seeding cells, TDM scaffolds, and an inductive alveolar fossa microenvironment, successfully forming root-like tissues with a pulp-dentin complex, and a PDL connecting a cementum-like layer with the host alveolar bone [152]. This bioroot complex performed the masticatory function and kept a stable structure for around three months after crown restoration [153]. Alternatively, eight weeks after *in situ* implantation of DFCs/TDM, it displayed a soft tissue clearance between the TDM and jaw bone similar to the native tooth root, consisting of dense and well-aligned collagen fibers, fibroblasts, and blood vessels beneficial for PDL formation [33]. Considering the scant sources of allogeneic TDM (aTDM), xenogeneic TDM (xTDM) was a possible substitute for aTDM but it caused osteolysis and resorption lacunae and led to regenerated root failure. The *tert*-butylhydroquinone (*t*BHQ), an antioxidant, can reduce osteolysis and osteoclastic resorption when added in xTDM/aDFCs scaffolds [154].

### 7.3. Periodontium Regeneration

Periodontal tissue destruction caused by periodontal diseases has become the main cause of tooth loss and a huge challenge in oral treatment. The connection between the tooth root and alveolar bone decreases obviously due to the damage of collagen fiber of PDL. In the current treatment for periodontal diseases, the application of alloplastic materials and autografts are dependent on autologous tissue grafts or artificial implants, which are limited as a result of insufficient biocompatibility, the risks of reinfection, and bone resorption [116]. As a consequence, cell-based techniques have been a new trend for periodontal regeneration [155]. As the vital precursor cells to form periodontal tissues in tooth development, DFCs are excellent potential resources for periodontium regeneration. Oshima et al. developed a novel fibrous-connected tooth implant using a HA-coated dental implant and DFCs, which successfully restored physiological functions of the tooth, including the ability to respond to mechanical stress and noxious stimulation, bone remodeling in severe bone defects [156]. Later, a multilayer construct emerged to induce simultaneous regeneration of PDL, cementum, and alveolar bone in periodontium repairment. A bilayered construct with DFCs consisting of a polycaprolactone (PCL) multiscale electrospun membrane and a chitosan/2 wt% CaSO_4_ scaffold regenerated PDL and alveolar bone separately, and it showed better protein adsorption beneficial for cell attachment and proliferation [157]. Trilayered nanocomposite hydrogel scaffolding, composed of chitin poly(lactic-*co*-glycolic) acid (PLGA)/nanobioactive glass ceramic (nBGC)/cementum protein 1 (CEMP1), chitin-PLGA/fibroblast growth factor 2 (FGF2), and chitin-PLGA/nBGC/platelet-rich plasma- (PRP-) derived growth factors acting as the cementum layer, PDL layer, and alveolar bone layer, respectively, achieved a complete healing with the formation of new cementum, fibrous PDL, and alveolar bone with well-defined trabeculae, which served as a good alternative regenerative approach for periodontal diseases [150].

### 7.4. Other Tissue Regeneration

DFCs are also an alternative source for the regeneration of other tissues in addition to the tooth and bone. Due to neural differentiation capability of DFCs, an approach utilized human DFCs (hDFCs) and aligned electrospun PCL/PLGA material (AEM) to reconstruct SCD, the developed oriented fibers *in vitro* and trend to differentiate an oligodendrogenic lineage in the SCD microenvironment may contribute to remyelination [158]. Alternatively, DFCs behaved a cardiomyogenic differentiation potential with the influence of SAHA *in vitro* and a small number of induced cardiomyocytes (iCMs) homed to the heart muscle without leading inflammatory or immune responses via systemic administration. However, the low homing ratio was unfavorable factors for standardized treatment [120].

DFCs, which possess multipotent differentiation ability and excellent immunosuppression capacities, are regarded as an alternative resource for repairing both hard tissue and soft tissue defects. However, the restoration of both morphology and function of damaged and infected tissues bring about enormous challenges. Quantities of researches were carried out to optimize the regenerative strategies depending on the rapid development of other subjects. In spite of a great prospect for DFCs in clinical application, the following points have been taken into account. Firstly, the requirement of donors including the age and health condition of periodontium should be emphasised since they impacted the regenerative properties of stem cells. Then, optimizing the strategy of DFC isolation and expansion *in vitro* beforehand is necessary. Proper heat stress conditions were beneficial to obtain the DFCs population containing more stem cells. Some isolation strategies such as the enzymatic digestion (EZ) and the outgrowth (OG) method did not affect DFCs-derived cell growth and isolated DFCs were capable of forming cementum-like matrix *in vitro* and acellular cementum structures *in vivo* [132, 159]. Besides, age-related cellular changes of DFCs regarding the loss of stemness and differentiation capability are expected to be improved [160]. More importantly, preclinical evaluations of dental stem cells especially on large animal models followed by randomized clinical trials are required [161]. Also, clinical trials evaluating DFC application in bone or tooth tissue engineering should be carried out to identify the actual feasibility of clinical application. Therefore, we are faced with the coexistence of opportunities and challenges and there is a long way to go.

## 8. Conclusion

In this article, we reviewed roles of DFCs in tooth development, the characteristics of DFCs including their multilineage differentiation, immunosuppressed capability, and excellent amplification ability and their tissue engineering potentials. Meanwhile, experimental or clinical application progresses on tissue regeneration such as the bone regeneration, dental root establishment, and periodontium recovery. Therefore, DFCs can act as a group of excellent cells for future cell-based treatment for tissue repairment and regeneration.

## Figures and Tables

**Table 1:** Comparison of tooth development-related mesenchymal stem cells/progenitor cells.

	Proliferation capacity	Cell surface markers		Multipotent differentiation in vivo/in vitro	Immunomodulatory properties	Potential clinical application	References
		Positive	Negative				
DFCs	++++	CD9 CD10 CD13 CD29 CD44 CD53 CD56 CD59 CD73 CD90 CD105 CD106 CD146 CD166 CD271 STRO-1 NOTCH-1 HLA-ABC, NANOG SOX2 OCT4, nestin, and beta-III-tubulin	CD31 CD34 CD45 CD133	Alveolar bone, PDL, cementum, adipocyte, osteoblast, cementoblast/chondrocyte, neuron-like cell cardiomyocyte, and dentin-like tissues	Immunosuppressive properties expressing TLR2, TLR3, and TLR4; increased IL-10, IL-6, TGF-*β*, and IDO-1; decreased IFN-*γ*, IL-4, and IL-8; suppressed proliferation of PBMCs; decreased number of CD4^+^ T cells and increased regulatory T cells	Bone defects, tooth root regeneration, periodontal tissue regeneration, and neural tissue regeneration	[11–16]

DPSCs	+++	CD9 CD10 CD13 CD29 CD44 CD59 CD73 CD90 CD105 CD146 CD106 CD146 CD166 CD271 STRO-1, TRA1-60, and NANOG SOX2 Oct-4 TRA-1-80-1	CD14 CD19 CD24 CD117 CD34 CD45 CD31 CD133	Adipocyte, dentin-pulp, bone muscle/odontoblast, myoblast adipocyte, osteoblast, neuron-like cell, cardiomyocyte, and hepatocyte-like cell	Immunosuppressive properties increased HGF, TGF-*β*, PGE-2, IL-6, IDO, and IL-10; decreased IL-4 and IFN-*γ*; increased number of regulatory T cells; suppressed proliferation of T cells and PBMCs; inducing activated T cells apoptosis	Bone defects, dentin-pulp repair, and neural tissue regeneration	[11, 15, 17–21]

PDLSCs	++	CD9 CD10 CD13 CD29 CD44 CD59 CD73 CD90 CD105 CD106 CD146 CD166 CD271 STRO-1	CD11b CD14 CD19 CD34 CD45 CD79*α* HLA-DR	Cementum, PDL/chondrocyte, osteoblast, cementoblast, and adipocyte neuron-like cell	Immunosuppressive properties expressing TLR2 and TLR4; released IDO, HGF, and TGF-*β*; suppressed proliferation of PBMCs and reduced induction of Tregs	Tooth root regeneration and periodontal tissue regeneration	[14, 15, 18, 21–24]

ABMSCs	+	CD13 CD29 CD44 CD71 CD73 CD90 CD105 CD146 CD166 STRO-1, OCT4 NANOG SOX2, and nestin	CD11b CD14 CD19 CD34 CD45 CD31	Bone/osteoblast, chondrocyte, and adipocyte	Expressing TLR2, 4 5, 7, 1, 10, 8, 3, and 6	Bone defect and periodontal regeneration	[15, 25–29]

SHEDs	+++	CD29 CD73 CD90 CD105 CD146 CD166 STRO-1, NANOG, and nestin	CD14 CD34 CD45	Bone dentin-pulp, microvessels/chondrocyte, myocytes, adipocytes, osteoblasts, and neuron-like cell	Immunosuppressive properties inhibited Th17 cell differentiation; increased number of Tregs; corrected CD4^+^ T cell immune imbalance in allergic diseases; increased IL-10 and decreased IL-4 and IFN-*γ*	Craniofacial bone defects, dentin-pulp repair, Neural regeneration, and tooth root regeneration	[11, 30–37]

SCAPs	++	CD13 CD29 CD49 CD51 CD56 CD61 CD73 CD146 CD90 CD44 CD24 CD106 CD146 CD166 STRO-1, NANOG, and nestin	CD14 CD18 CD34 CD45 CD117 CD150	Dentin-pulp/osteoblast, adipocyte, odontoblasts, hepatocytes, and neuron-like cell	Low immunogenicity inhibited proliferation of T cells; overexpressed Nfic to suppress LPS-initiated innate immune responses	Bone regeneration, tooth root regeneration, dentin-pulp repair, neural regeneration and repair, and periodontal regeneration	[30, 31, 38–41]

GMSCs	++	CD29 CD44 CD73 CD90 CD105 CD106 CD166 STRO-1, NANOG, and nestin	CD14 CD117 CD34 CD45	Cartilage, bone, muscle/adipocyte, chondrocyte, osteocyte, and neuron-like cell	Immunosuppressive properties expressing TLR1, 4, 5, 7, and 10; inhibited proliferation of T cells, Th17; increasing CD4^+^ CD25^+^ FoxP3^+^ Tregs; releasing IL-6, IDO, IL-10, COX-2, and iNOS	Calvarial defects, neural regeneration, and periodontal regeneration	[32, 42–45]

TGSCs	Not reported	CD29 CD73 CD90 CD105 CD166 STRO-1	CD14 CD34 CD45 CD133	Bone/osteoblast, odontoblast, adipocyte, chondrocyte, and endothelial cell	Seldom reported	Bone repair and cartilage regeneration	[30, 46, 47]

DPSCs: dental pulp stem cells; PDLSCs: periodontal ligament stem cells; ABMSCs: alveolar bone-derived mesenchymal stem cells; SHEDs: stem cells from exfoliated deciduous teeth; SCAPs: stem cells from apical papilla; GMSCs: gingiva-derived mesenchymal stem cells; TGSCs: tooth germ stem cells.
